# Activate or Inhibit? Implications of Autophagy Modulation as a Therapeutic Strategy for Alzheimer’s Disease

**DOI:** 10.3390/ijms21186739

**Published:** 2020-09-14

**Authors:** Sharmeelavathi Krishnan, Yasaswi Shrestha, Dona P. W. Jayatunga, Sarah Rea, Ralph Martins, Prashant Bharadwaj

**Affiliations:** 1 Centre of Excellence for Alzheimer’s Disease Research and Care, School of Medical and Health Sciences, Edith Cowan University, Joondalup, WA 6027, Australia; skrishn3@our.ecu.edu.au (S.K.); yasaswis@our.ecu.edu.au (Y.S.); d.jatatunga@ecu.edu.au (D.P.W.J.); r.martins@ecu.edu.au (R.M.); 2 Harry Perkins Institute of Medical Research, Centre for Medical Research, University of Western Australia, Nedlands, WA 6009, Australia; Sarah.Rea@murdoch.edu.au; 3 Perron Institute for Neurological and Translational Science, Centre for Neuromuscular and Neurological Disorders, The University of Western Australia, Nedlands, WA 6009, Australia; 4 Centre for Molecular Medicine and Innovative Therapeutics, Murdoch University, Health Research Building, Discovery Way, Murdoch, WA 6150, Australia; 5 School of Biomedical Science, Macquarie University, Sydney, NSW 2109, Australia; 6 Alzheimer’s Australia Research Foundation, Nedlands, WA 6009, Australia; 7 School of Pharmacy and Biomedical Sciences, Curtin Health and Innovation Research Institute (CHIRI), Faculty of Health Sciences, Curtin University, Bentley, WA 6107, Australia

**Keywords:** beta amyloid, autophagy, clearance, toxicity, lysosome, Alzheimer’s disease, ageing, stress response, apoptosis

## Abstract

Neurodegenerative diseases result in a range of conditions depending on the type of proteinopathy, genes affected or the location of the degeneration in the brain. Proteinopathies such as senile plaques and neurofibrillary tangles in the brain are prominent features of Alzheimer’s disease (AD). Autophagy is a highly regulated mechanism of eliminating dysfunctional organelles and proteins, and plays an important role in removing these pathogenic intracellular protein aggregates, not only in AD, but also in other neurodegenerative diseases. Activating autophagy is gaining interest as a potential therapeutic strategy for chronic diseases featuring protein aggregation and misfolding, including AD. Although autophagy activation is a promising intervention, over-activation of autophagy in neurodegenerative diseases that display impaired lysosomal clearance may accelerate pathology, suggesting that the success of any autophagy-based intervention is dependent on lysosomal clearance being functional. Additionally, the effects of autophagy activation may vary significantly depending on the physiological state of the cell, especially during proteotoxic stress and ageing. Growing evidence seems to favour a strategy of enhancing the efficacy of autophagy by preventing or reversing the impairments of the specific processes that are disrupted. Therefore, it is essential to understand the underlying causes of the autophagy defect in different neurodegenerative diseases to explore possible therapeutic approaches. This review will focus on the role of autophagy during stress and ageing, consequences that are linked to its activation and caveats in modulating this pathway as a treatment.

## 1. Introduction

With ageing, there is a decline in the cell’s ability to maintain protein homeostasis, or proteostasis, and this natural decline is characteristically exacerbated in all neurodegenerative diseases [1]. Cell survival, growth and proliferation rely on a balance between protein synthesis, folding, trafficking, aggregation and degradation. The cellular maintenance of proteostasis involves controlling the conformation, binding interactions, location and concentration of individual proteins making up the proteome [1]. Protein folding is accomplished through interactions between the folding polypeptide chain and macromolecular cellular components, including multiple classes of chaperones, folding enzymes and targeted degradation pathways, which minimize protein aggregation [2]. Human loss of function diseases are often the result of a disruption to normal proteostasis, typically caused by mutation in a related gene, thereby compromising the protein folding. In contrast, gain of function diseases may occur as a result of disrupted proteostasis leading to decreased ability to degrade misfolded proteins that results in accumulation of toxic protein aggregates, such as beta-amyloid (Aβ) in Alzheimer’s disease (AD) [3]. Macroautophagy is a bulk-degradation mechanism of autophagy that is induced by starvation and is important for the clearance of protein aggregates and the removal of damaged organelles. Macroautophagy aids to indemnify the lack of nutrients by regenerating the availability of building units non-selectively [4]. However, autophagy can also be highly selective for damaged organelles and specific proteins and is an important therapeutic approach for protein misfolding diseases like AD. Autophagy activation is; therefore, an attractive therapeutic strategy for AD. However, excessive stimulation of this pathway can be detrimental and potentially lethal to cells with underlying dysfunctional proteostasis. Here, we review the dual role of autophagy as a protective pathway regulating proteostasis and its pathogenic role in promoting neurodegeneration in AD. We also discuss whether activation or inhibition of the autophagic pathway has the best potential as a therapeutic strategy in AD.

## 2. Autophagy Process

Autophagy can be classified as selective or non-selective. Selective autophagies include mitophagy (mitochondria), ribophagy (ribosome), lysophagy (lysosomes), pexophagy (peroxisomes), lipophagy (lipid droplets), glycophagy (glycogen), aggrephagy (misfolded proteins), xenophagy (infected pathogens) and reticulophagy (endoplasmic reticulum) [5]. Autophagy “cargo” receptor like p62 recognizes specific cargo for autophagic degradation [6]. Conversely, non-selective autophagy involves the digestion of random parts of the cytoplasm and its components; a process that maintains proteostasis [7].

Autophagy maintains cellular homeostasis and is usually induced under stress conditions such as nutrient starvation, the presence of unfolded proteins, viral infection or oxidative stress. Nutrient signalling is regulated by mTOR (mammalian target of rapamycin) and therapeutic strategies that involve direct or indirect inhibition of mTOR, such as CCI-779 or Rapamycin. However, deprivation of amino acids can promote mTOR-independent autophagy proteolysis [8,9]. Deficiency of certain amino acids can stimulate autophagy but is dependent on cell type and the type of amino acids which are lacking. Additionally, other factors can affect autophagy such as AMP-activated protein kinase (AMPK) [10,11] and Bcl-2 (B-cell lymphoma 2) [12]. 

**Figure 1:**
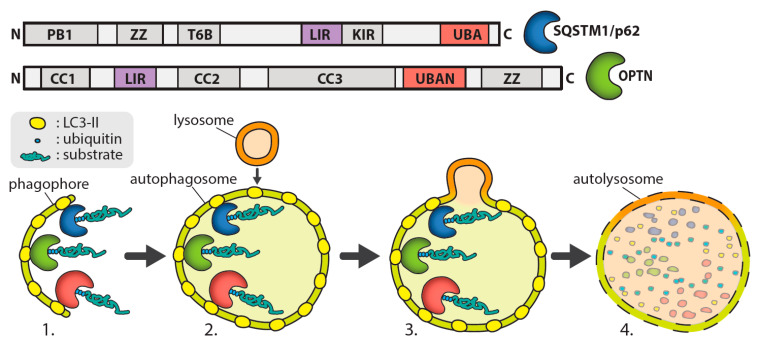
**Formation of phagophore in autophagy.** The above diagram shows the formation of the double-membraned structure during Macroautophagy. Domains of SQSTM1/p62; PB1 (Phox and Bemp1), ZZ (zinc finger), T6B (tumour necrosis factor receptor associated factor 6 binding), LIR (LC3 interacting region), KIR (Kelch-like ECH associated protein 1 interacting region), UBA (ubiquitin-associated). Domains of OPTN; coiled coil (CC1-3), LIR, UBAN (ubiquitin-binding domain in ABIN proteins and NEMO), ZZ (ZZ-type zinc finger).

In brief, the autophagy process involves the formation of an autophagosome, which encapsulates a section of cytoplasm sequestering misfolded proteins, long-lived proteins, and organelles and then fuses with lysosomes to enable substrate degradation. Autophagosome formation involves the initiation of a double membrane organelle called a phagophore that surrounds the cytoplasm component, including organelles [12], as shown in Figure 1. The autophagosome then fuses with lysosomes, leading to the hydrolyzation of the inner membrane of the autophagosome and release the cytoplasm-derived components. Degraded macromolecules are then transported back to the cytosol to be reused [12]. In selective autophagy, autophagy receptors such as optineurin or p62 recognise ubiquitinated proteins for degradation via their UBAN (ubiquitin-binding domain in ABIN proteins and nemo) or UBA (ubiquitin-associated) domains, respectively, and traffic them to the phagophore [13]. The autophagy receptors then bind to the autophagosomal membrane through an interaction with light chain 3 (LC3) mediated by an LC3-interacting region (LIR). 

Evidence for the important role of autophagy in maintaining proteostasis in the CNS is demonstrated by studies showing that autophagy dysfunction contributes to the accumulation of misfolded proteins, including aggregation of hyper-phosphorylated tau and Aβ leading to neurodegeneration in AD [14,15]. Inhibition of crucial autophagy genes such as Atg7 or Atg5 lead to different consequences depending on whether inhibition is complete or selective. Studies showed that complete inhibition caused mortality in newborn mice, while selective inhibition led to phenotypes that are reminiscent of neurodegenerative diseases [15]. Inhibition of chaperone-mediated autophagy (CMA) contributes to the development of neurofibrillary tangles made up of hyper-phosphorylated tau proteins. The tau proteins which are bound to lysosome-associated membrane protein type 2a (LAMP-2A) not only affect lysosomal membranes, but also hinder the mechanism of CMA [16]. Increased levels of regulator of calcineurin 1 (RCAN1) in the brain further demonstrates CMA disruption in AD [17]. 

## 3. Role of Autophagy as a Stress Response Pathway

**Figure 2:**
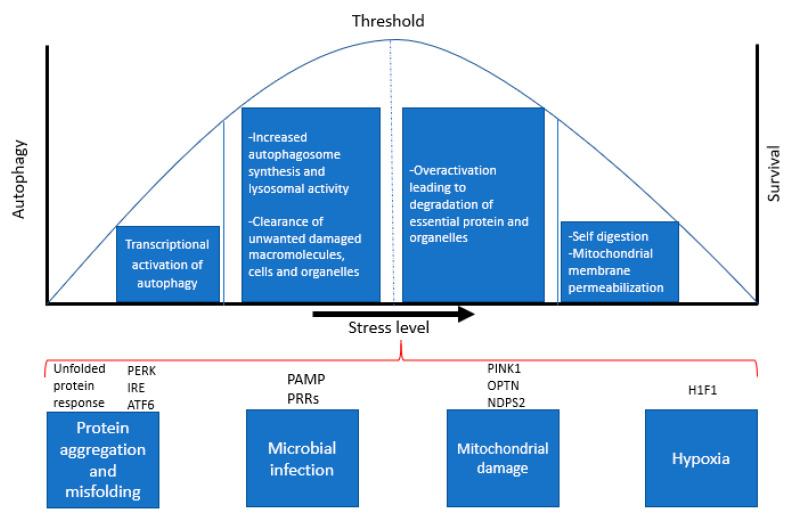
**Cellular stress response pathways and regulation of autophagy and apoptosis**. The above diagram is a representation of various stress responses that activate autophagy and indicates the levels of stress that determines the cell’s ability to survive or undergo apoptosis.

Eukaryotic cells must adapt continuously to changes in external conditions that induce stress, including temperature, ion concentrations, oxygen levels, pH and microbial pathogens. More importantly, cells need to maintain intracellular homeostasis by constantly removing unwanted macromolecules and cellular waste, accumulation of which is a hallmark feature of AD and many neurodegenerative diseases. Beyond a certain threshold, such changes become “stressors”, meaning that the cellular response to this stress determines whether the cell can function effectively and survive (Figure 2). Autophagy constitutes an important protective mechanism that promotes cell survival in response to multiple stressors and helps defend against degenerative, inflammatory and infectious diseases [18,19]. Autophagy can be induced by a variety of stress stimuli, including nutrient and energy stress, proteotoxic stress, pathogen-associated molecular patterns (PAMPs), hypoxia and mitochondrial damage [20]. 

Nutrient depletion or starvation is the most potent physiological inducer of autophagy [21,22]. Several critical molecules regulate starvation-induced autophagy; of these, nutrient signalling pathways via the kinases mTOR and AMPK are the best characterized. The unfolded protein response (UPR) [23] is a potent stimulus of autophagy in response to protein aggregation and toxicity. The UPR is mediated by PERK (PKR-like eIF2a kinase), ATF6 (activating transcription factor-6), and IRE1 (inositol requiring enzyme 1), all of which are regulated by the chaperone binding immunoglobulin protein (BiP/GRP78). BiP/GRP78 binds to misfolded proteins, thereby releasing PERK, IRE1, and ATF6 from their inhibitors, resulting in autophagy activation. Among these, PERK and ATF6 act as autophagy inducers, while IRE1 acts as a negative regulator of autophagy [24].

Microbial infection constitutes a specialized form of cellular stress that results in autophagy induction [25]. Autophagy activation during infection is regulated by cytokines such as interferon gamma (IFN-γ) and pathogen recognition receptors (PRRs) that recognize conserved components of pathogens (PAMPs) [26]. In addition, PRRs recognize PAMPs, necrotic cells, hypoxia, reactive oxygen species (ROS), and the accumulation of misfolded proteins. Hypoxia (with oxygen concentrations < 3%) also induces autophagy through a variety of different mechanisms. Hypoxia-induced autophagy depends on hypoxia-inducible factor (HIF), while anoxia-induced autophagy is HIF-independent [27,28].

Mitochondrial damage is another potent inducer of autophagy and considerable advances have been made in understanding the mechanisms by which damaged mitochondria are targeted for autophagy, and the functional significance of mitochondrial quality control in preventing ageing, neurodegenerative diseases and other pathologies. Cells clear damaged mitochondria via mitophagy to prevent the accumulation of ROS. The autophagic recognition of damaged mitochondria is mediated by the mitochondrial kinase, PINK1 [29], which also plays a critical role in maintaining mitochondrial integrity [30]. The upregulation of autophagy by these stimuli involves diverse signals that often have overlapping functions in autophagy, cellular stress responses and cell death pathways, which is discussed in the next section. 

## 4. Dual Role of Autophagy: Crosstalk between Autophagy and Apoptosis

Autophagy is constitutively active in the central nervous system (CNS) [31] and helps maintain homeostasis by eliminating defective proteins and organelles, preventing the accumulation of protein aggregates, maintaining energy demands, and supporting neuronal plasticity [32,33]. Evidence indicates that autophagy is neuroprotective [34], which is particularly important in post-mitotic cells like neurons [13,35]. Neurons have many specialized cell processes for neurotransmission including axons and synapses that require high energy and protein turnover. Autophagy vesicle trafficking in neurons can be very lengthy, as autophagosome formation occurs in the distal axon which undergoes retrograde trafficking to the soma. Therefore, neurons are particularly sensitive to defects in autophagy-lysosomal trafficking and axonal injuries that induce the accumulation of autophagosomes or autophagic vesicles (AVs) [36].

Autophagy is closely related to programmed cell death or apoptosis, which is primarily initiated by mitochondrial membrane permeabilization (MMP) [31]. Selective autophagic removal of depolarized mitochondria or mitophagy occurs if a small fraction of mitochondria displays MMP. However, beyond a certain threshold for mitophagy, which varies between cell types and the nature of the stress involved, MMP constitutes an irreparable and lethal event. It is plausible that liberation of the apoptosis regulator protein Bcl-2 from activated autophagy protein complexes may free up these molecules to block pathways of apoptosis [17]. Considering the predominantly cytoprotective role of autophagy, it seems likely that apoptosis induction would be coupled to autophagy inactivation. For example, Caspase-3 cleaves Beclin-1, thereby destroying its proautophagic activity. The C-terminal fragment of Beclin-1 that results from this cleavage acquires a new function and can amplify mitochondrion-mediated apoptosis [37]. Caspase-3 activation also cleaves and activates Atg4D, an enzyme that catalyses the delipidation of the LC3 paralog GABARPL1. This proteolytic activation increases Atg4D recruitment to mitochondria via a putative BH3 domain and enhances its cytotoxic activity [38]. Similarly, the proteolytic activity of calpain can destroy the proautophagic function of Atg5 [39], generating a proapoptotic mitochondrion-permeabilizing Atg5 fragment [40]. 

Although autophagy is generally considered a pro-survival mechanism, autophagy and apoptosis are interdependent. Autophagy impairment causes an increase in neuronal apoptosis [41]. Autophagy has been associated with promotion of cell death as a result of excessive activation [42] and also during cell elimination and neuronal excitotoxicity [43,44,45]. Some studies have shown that autophagy inhibition increases neuronal survival in cases such as hypoxic/ischemic brain injury in mice and necrotic cell death in *Caenorhabditis elegans* [46,47,48]. It is likely that excessive autophagy activation and long-term autophagy up-regulation eventually results in self-digestion [49,50,51]. The role of autophagy in cell death and the detailed mechanisms involved are still unclear and it is debated whether autophagic cell death is apoptosis-related or is a separate process [18,34,52]. Of note, apoptosis and autophagy have shared regulators, including Beclin-1, Bcl-2, p53, and Atg5, which may interact to promote neuronal cell death [19,53]. Collectively, these findings underscore that autophagy and apoptosis are interdependent with multiple shared signalling pathways and regulatory processes.

## 5. Autophagy and Ageing

Age is the most important risk factor for AD. Ageing is a natural event occurring in all living organisms, and constitutes a decline in repair processes including autophagy, leading to damage accumulation and progressive deterioration of cell function. Many organisms show signs of decreased autophagic capacity with age and growing evidence supports its role as an anti-ageing mechanism. Decreased autophagy and lysosomal proteolysis during ageing has been extensively reported (reviewed in [54]). Electron microscopy studies and metabolic assays revealed that rates of proteolysis and sensitivity to autophagy stimulation decline with age ([55,56]). The regulation of autophagy by hormones is also differently affected by age ([57]). Lysosomes isolated from different tissues of aged rodents had reduced ability for binding and uptake of the cytosolic substrate proteins. Interestingly, the degradation of the substrates was unperturbed by age, suggesting that the activity of the lysosomal enzymes is preserved during ageing ([58]. CMA also declines with age. Reduction of LAMP-2A receptors, which are essential in the mechanism of CMA, is said to affect the rate of proteolysis and cytoprotective function due to the interruption in the binding of the substrate to the lysosomes. Furthermore, cholesterol levels in the lysosomal membrane are affected by the level of LAMP-2A genes [59]. The efficiency of transcription factors linked with autophagy, such as transcription factor EB (TFEB), also contributes to the decline of autophagy due to ageing [60]. A recent study shows that overexpression of TFEB results in longevity [59].

Numerous studies involving knockout (KO) or induction of autophagic genes have further revealed the importance of autophagy in ageing [61]. Atg5 overexpression increased lifespan along with enhanced autophagy, leanness, insulin sensitivity and motor function in Atg5 transgenic mice [62]. Reducing the decline in levels of LAMP-2A preserved autophagic activity and was associated with reduced accumulation of damaged proteins and improved organ function in mice [63]. In a nematode model, Caenorhabditis elegans [64], restoring expression of Atg18 in neurons of Atg18; daf-2 double mutants fully rescued the shortened lifespan of these animals [65]. Furthermore, several autophagy transgenic mouse models have an extended lifespan, although the molecular mechanisms behind this and the connection with ageing are unclear [66]. Tissue-specific ablation of essential Atg genes shows premature signs of ageing and specific-Atg5 or Atg7 KO leads to neurodegeneration [67]. The level of expression of certain genes plays a crucial role in the regulation of autophagy and lifespan extension. Overexpression of sirtuin 1 (SIRT1) activates autophagy, delays ageing and promotes longevity in both cell and animal models [59]. Furthermore, clinical findings show that serum concentrations of Beclin-1, a key regulator of autophagy, is associated with longevity in humans [68]. In this study, healthy centenarians had significantly higher Beclin-1 levels compared with young subjects, suggesting that elevated basal levels of autophagy may be a biomarker of longevity in humans.

In addition to lifespan extension, studies have also linked autophagy to improved cognitive functions [69,70,71]. Many studies have investigated neurodegenerative diseases related to ageing with autophagy being the main risk factor [72,73]. Most neurodegenerative diseases share the characteristic of misfolded proteins and damaged organelles, and these accumulations interfere with proper axonal trafficking, culminating in neurotoxicity. Impairment of either autophagy or CMA hampers the remodelling of dendrites and axons, thus diminishing neuronal plasticity [74,75]. In AD, extracellular Aβ plaques secreted by autophagosomes can interrupt intercellular communication [75]. Age-related decline in memory formation is well established and the above findings support a model in which a decline in autophagy contributes to the cognitive loss associated with ageing. But the underlying mechanisms of how autophagy may contribute to brain function during ageing is not well understood. It is plausible that autophagy is important for many processes in the CNS that contribute to ageing, including clearance of long-lived and aggregate-prone cytoplasmic proteins and damaged organelles, to preserve neuronal integrity and promote survival during ageing.

## 6. Role for Autophagy Genes in AD

Apolipoprotein E4 (ApoE4) is the main genetic risk factor for sporadic AD [74,76]. ApoE4 is vital for lipid homeostasis, which also extends to autophagy mediated clearance; however, despite several decades of research, the exact mechanisms underlying its contribution to AD pathogenesis remain incompletely defined. Genetic risk factors that alter autophagic processes are also implicated in other neurodegenerative diseases (e.g., Huntington’s disease, amyotrophic lateral sclerosis and frontotemporal degeneration) [77]. In all of these cases, the lack of efficient degradation by autophagy leads to the prolonged presence of aggregated proteins that elicit axonal transport malfunction [78]. 

Mutations within autophagy receptor genes, *SQSTM1*, *OPTN* and *UBQLN2*, and autophagy regulator genes, *VCP* and *TBK1*, are associated with frontotemporal degeneration and amyotrophic lateral sclerosis [79]. Of these, mutations in *SQSTM1* have also been reported in AD cases [80]. Following a meta-analysis that revealed the subgenome-wide association of a *SQSTM1* intronic variant with AD, targeted sequencing was performed on a Flanders-Belgian cohort of patients with either early onset AD (EOAD) or a positive family history of AD. A total of 61 *SQSTM1* exonic variants were reported, 57 of which were rare variants. While rare variant burden analysis did not reveal an increased frequency in patients compared to controls, two common synonymous variants showed a nominal association with AD [80]. Targeted resequencing of the *TBK1* gene in 1253 EOAD patients and 2117 control individuals revealed 32 rare variants, one of which was a loss-of-function mutation. Of the 31 missense variants identified, seven were exclusive to patients and four of these had combined annotation dependent depletion (CADD) Phred scores of >20, which can be indicative of pathogenicity. However, control-specific and shared variants also attained high (>20) CADD Phred scores and no enrichment of rare variants in cases compared with controls was observed [81]. There have been no reports of an association of *OPTN* or *UBQLN2* variants with AD. However, a recent study identified a variant of unknown significance in *VCP* in an EOAD patient [82]. Transcript analysis from the cerebellum and temporal cortex of AD patients highlighted that an *OPTN* single nucleotide polymorphism previously associated with Paget’s disease of bone was linked with increased OPTN expression [83]. Overall, a significant role for autophagy receptor genes or their regulators in AD is not supported. 

A recent review of the gene associations reported in AD highlighted the potential or confirmed involvement of >40 genes [84] and some of these have roles in autophagy. TREM2 has been shown to enhance microglial metabolism via regulation of mTOR [85]. Silencing of *EPHA1* blocks autophagy [86]. Clusterin, a chaperone involved in proteostasis, is required for pro-survival autophagy [87], LC3 lipidation and autophagosome biogenesis [88]. PICALM modulates autophagy, and subsequently tau turnover via endocytic trafficking [89]. KAT8 is a histone acetyltransferase, overexpression of which increases autophagic flux [90]. WWOX (WW domain containing oxidoreductase) suppresses autophagy via a direct interaction with mTOR that leads to a reduction in Beclin-1, Atg12-Atg5 and LC3-II levels [91]. Lastly, MAPT/tau has been shown to repress autophagosome-lysosome fusion by impeding ESCRT-III complex formation and this leads to an accumulation of LC3-II, p62 and autophagosomes [92]. 

## 7. Role of Autophagy in AD Pathogenesis

Senile plaques containing Aβ protein and neurofibrillary tangles (NFTs) composed of the hyper-phosphorylated tau protein are the pathological hallmarks of AD [93]. Senile Aβ plaques are specific for AD, whereas NFTs are found in several tauopathies and are a common marker of neurodegeneration. Aβ is an amyloidogenic protein produced by amyloid precursor protein (APP) through proteolytic cleavage at the N- and C-termini by β-secretase and γ-secretase, respectively [94]. The number of amino acid residues in Aβ can range from 39 to 43, with Aβ40 being the most predominant species [95]. However, Aβ42 tends to be the main toxic species and protein component in senile plaques in the AD brain [96]. In the brain, Aβ42 is present in low levels during normal physiological conditions. However, in pathological conditions, Aβ42 is present in high concentrations and adversely affects neuronal function. Aβ42 is highly aggregate prone and can form multiple isoforms ranging from small oligomers to mature fibrils. Growing evidence shows that soluble Aβ42 oligomers are the main toxic species, while plaques (which mostly contain insoluble fibrils) are considered relatively inert, secondary contributors to the onset of cellular dysfunction and sometimes even a protective mechanism to limit oligomer toxicity [97].

Dysfunctional autophagy is implicated in the disruption of cell signalling pathways [98] and increased accumulation of Aβ and tau protein aggregates in AD [99,100]. In contrast to normal ageing, excessive accumulation of autophagy vesicles (AVs, autophagosomes and lysosomes) has been observed in post-mortem brain of AD patients [101,102,103], raising the question of whether the AV accumulation is a result of autophagy dysfunction [102,104] or due to excessive autophagy [105]. Autophagy is regulated by the nutrient signalling kinase AMPK, and its dysregulation is proposed to contribute to autophagy dysfunction and neurodegeneration in AD [106]. AMPK is a heterotrimeric protein complex composed of 3 subunits including a non-catalytic regulatory gamma subunit PRKAG2 (protein kinase AMP-activated non-catalytic subunit gamma 2) that modulates the ability of the kinase to control autophagy during stress. The PRKAG2 homolog, SNF4Aγ is required for regulation of developmental and stress-induced autophagy in a fruit fly model of AD [107]. Recent findings from our group show that expression of the PRKAG2 gene was increased three-fold in AD-hippocampus and the AD-frontal cortex, and its protein levels positively correlated with Aβ accumulation in the brain [108]. In yeast, we showed that Aβ42 expression activates autophagy and co-expression of SNF4 (PRKAG2 homolog) markedly reduced the levels of Aβ42 aggregates and autophagic activity [109]. Overall, these findings show that increased autophagy activation and expression of PRKAG2 could be a response to increased Aβ accumulation in the AD brain. Our studies also suggest that reduced activation of genes like PRKAG2 maybe an important contributor to Aβ accumulation and neurodegeneration in AD.

The equilibrium between the formation of new autophagosomes, maturation and clearance of autophagosomes by lysosomal degradation is known as autophagic flux and is becoming an increasingly important concept in understanding neurodegenerative disease pathogenesis. The constant presence of AV’s in neurodegenerative states indicates induction of autophagy [110]. Newly-formed autophagosomes are usually eliminated in the neurons by fusion with lysosomes to prevent the build-up of autophagic intermediates, which are not normally found in a healthy brain [111,112]. The clearance of AVs can be obstructed by inhibition of autophagosome–lysosome transport or by blocking substrate proteolysis, which results in a massive accumulation of AVs in the AD brain and in PS/APP, a double transgenic model that expresses familial AD mutation in Presenilin-1, PS1(M146L) [110].

In addition to excessive autophagy, evidence shows impaired maturation or fusion of autophagosomes with lysosomes or their transport toward the neuronal cell body in AD. Dysfunction of endosomal–lysosomal trafficking is also observed with accumulation of AVs in post-mortem human brain and in a mouse model of AD [112,113]. A study in mouse blastocysts has shown that a mutation in presenilin-1 (PS1) caused lack of Aβ clearance. PS1 is required for lysosomal turnover of autophagic and endocytic protein substrates, and its deletion caused loss of autophagy function due to impaired autolysosome acidification and cathepsin activation (cysteine cathepsins are proteases responsible for proteolytic degradation within the lysosome) [113,114]. Accordingly, enhancing lysosomal cathepsin activity in an AD mouse model reduced the accumulation of ubiquitinated proteins and other autophagic substrates within AVs, and decreased extracellular and total brain Aβ deposition. Overall, these studies suggest that prominent lysosomal dysfunction is present in AD. In AD mouse models, autophagosome accumulation in dendrites precedes extracellular plaque formation, indicating that autophagy is an early response and not as an outcome of plaque formation during end-stage disease [115]. Furthermore, APP, PS1 and other substrates necessary for the generation of Aβ peptides were identified in isolated AVs in the livers of AD mice [114], suggesting a potential role for AVs in Aβ generation [116]. A study in AD patients’ mid-frontal cortex grey matter found a reduction of Beclin-1 protein levels. Moreover, in an AD mouse model, reduction of Beclin-1 expression resulted in increased intraneuronal and extracellular Aβ accumulation, neuronal abnormalities and neurodegeneration [117], further supporting a role for inhibited autophagy in AD pathogenesis.

Microglial cells represent the immune system in the CNS. Microglia are located throughout the brain and spinal cord, accounting for 10–15% of all cells found within the CNS [118]. In addition to being the initial and primary form of defence mechanism in the brain that scavenges for plaques, damaged neurons and microbes, microglia are critical for overall brain maintenance and synaptic pruning during development. The observation of activated microglia around senile plaques in AD brain is well documented [119,120]. Autophagy in the CNS has been studied mainly in neurons but remains largely unexplored in other cell types including microglia. Glial cells can internalize Aβ and are implicated in its clearance [121,122,123,124]. Aβ exposure activates astrocytes, an abundant glial cell, however, chronic exposure may have detrimental consequences by over-activating autophagy, inducing release of glial inflammatory cytokines and nitric oxide that leads to neuronal and glial cell death [125]. A recent study reported changes in glia and autophagy in the hippocampus of AD mice at different stages of Aβ pathology. Interestingly, changes in microglial morphology were observed before Aβ plaque deposition and increased autophagy in glial cells was associated with Aβ deposition [126]. Astrocytes around plaques showed increased LC3. Moreover, Iba1 (ionized calcium-binding adaptor molecule 1), an inflammatory marker was co-localized with ubiquitin or p62 and was exclusively found in microglia [126]. Overall, these findings show that disturbed autophagy in glia is an early event that precedes Aβ plaque deposition. Therefore, particular attention to the timing of a possible intervention should be taken into consideration.

## 8. Autophagy Therapeutics for AD

Chronic ageing diseases like AD favour the formation of a molecular and cellular environment that promotes dysfunction in protein degradation mechanisms and, thereby, accumulation of aggregate-prone proteins such as Aβ and tau. As summarized above, AD exhibits characteristics of aberrant autophagy which is postulated to be a central pathophysiological factor. The consequences of autophagic failure are different depending on the stage at which it occurs. It might occur at the level of autophagosome formation, resulting in accumulation of discarded cargo such as misfolded proteins and/or dysfunctional organelles [1,31,127], or in the failure to recognize autophagic cargo. The outcomes; however, may be the same, and depends on the extent of the recognition of dysfunction and the type of cargo. A third level of defect might arise if the autophagosomes are not properly cleared, leading to their accumulation. This could interfere with intracellular trafficking and result in the neuronal loss seen in AD [113]. 

Modifying autophagy pharmacologically is an attractive approach to prevent or to halt AD by enhancing the removal of aggregated proteins, thus protecting the cell from dysfunction and death [128]. However, identifying the specific autophagy failure is imperative to the development of therapeutic strategies [129]. Induction of autophagy reduces the levels of both the soluble and aggregated species in AD models and is associated with beneficial effects [130,131,132,133,134,135]. Many autophagy enhancing molecules have been developed [76,77] and their therapeutic effects have been extensively reviewed previously [136,137]. Autophagy inducers can be classified into two main groups: mTOR-dependent or mTOR-independent. mTOR inhibitors are either ATP-competitive inhibitors (e.g., Torin1) or non-ATP-competitive inhibitors (e.g., rapamycin and rapalogs) [138]. Because of their inhibition of mTORC1, mTORC2, and, in some cases, phosphoinositide 3-kinase (PI3K) activities, chronic dosing of ATP-competitive inhibitors of mTOR activity in animals presents significant toxicity issues [138]. However, non-ATP-competitive inhibitors like rapamycin and its analogues have shown benefits as autophagy inducers in animal models of AD, Parkinson’s disease (PD), Huntington’s disease (HD) and prion protein (PrP) disease [127,133,139,140,141,142]. They have relatively safer profiles due to their non-ATP competitive mode of action and selectivity for mTORC1 [143], In fact, everolimus, a rapalog, was recently approved by the Food and Drug Administration (FDA) for the treatment of tuberous sclerosis.

Many mTOR-independent autophagy activators target AMPK. Trehalose, a widely studied autophagy inducer in neurodegeneration models [66], has been characterized as an AMPK activator [144]. The molecular targets of trehalose may be GLUT proteins, a family of glucose transporters whose inhibition results in activation of AMPK [144]. Trehalose dosing in mice has shown therapeutic effects, concomitant with autophagy induction, in a wide range of neurodegenerative disease models, including AD, PD, frontotemporal lobar dementia (FTLD), HD, SCA17, PrP and amyotrophic lateral sclerosis (ALS) [145]. Metformin is another AMPK-dependent autophagy inducer that has shown beneficial effects in animal models of neurodegeneration including AD, HD, and Lafora disease [146].

A growing number of autophagy inducers have been identified, that may act on other pathways, including cyclic AMP (cAMP)/inositol triphosphate (IP3), such as rilmenidine, clonidine, minoxidil and verapamil [102]. Our group previously demonstrated that a small molecule antihistamine drug, latrepirdine, activates autophagy and reduces Aβ pathology in yeast and animal models of AD and PD [147,148,149]. More recently, we developed the use of a rapid absorbance-based assay to measure Aβ42 toxicity in yeast [148]. Here, we showed significant differences in the levels of protection against Aβ42 toxicity conferred by physiological (nitrogen starvation) and chemical inducers (latrepirdine, rapamycin and small molecule enhancer of rapamycin 28) of autophagy. In summary, our findings provide evidence for autophagy induction as a preventative treatment against oligomer Aβ42-mediated cell death and neurodegeneration in AD.

Autophagic flux is generally defined as a measure of autophagic degradation capacity and is increasingly considered to be a critically important concept for development of therapies for AD. However, despite significant advances in measuring different molecular aspects of the autophagic machinery, it has remained a challenge to measure autophagic flux in a reliable, sensitive and quantifiable manner. Additionally, it should be noted that autophagosome flux may be different from substrate clearance flux, since cargo import and degradation in lysosomes can also be facilitated by other selective pathways like CMA. Additionally, the protein expression levels of the cargo protein may be affected over time and is possibly dependent on free amino acid generation by lysosomal degradation. Although interventions such as rapamycin, trehalose and lithium are used in various disease models, differences in cell types, treatment concentrations and duration make the interpretation of the effects of these drugs on autophagic flux challenging [150]. For a clear understanding of the autophagy dysfunction in AD and for development of effective autophagy-based therapies, it is critical to be able to monitor autophagy activity in real-time and also measure biomarkers that can be applied in clinical settings to assess the therapeutic efficiency of autophagy modulation. Several methods have now been developed to reliably measure autophagy in mammalian cells. In summary, this will include immunoblotting analysis including (1) detection of the conversion of LC3-I (cytosolic form) to LC3-II (membrane-bound lipidated form) and p62, (2) detection of LC3 turnover by the comparison of two samples with and without lysosomal inhibitors and microscopy methods, including (3) detection of autophagosomes and autolysosomes by electron microscopy, (4) detection of GFP-LC3 (or endogenous LC3) puncta formation assay for counting the average number of punctate structures per cell by fluorescence microscopy, and (5) detection of the GFP fragment generated by the degradation of GFP-LC3 inside autolysosomes by immunoblotting with an anti-GFP antibody [151]. Methods for monitoring autophagy in mammalian cells has improved significantly in the recent decades but still presents major challenges. Firstly, there is the challenge of quantitatively measuring a dynamic process and the inherent limitations associated with inferences based on end-point measurements. Secondly, there has been the challenge of autophagy organelle heterogeneity (i.e., the occurrence of multiple autophagy derived organelles in different stages of maturation), which makes it very difficult to examine them accurately.

## 9. Conclusions

Autophagy plays a key role in maintaining cellular homeostasis and survival by promoting clearance of mutant/misfolded proteins. Despite significant progress in understanding the molecular and cellular mechanisms of autophagy, it is still unclear under which circumstances “enhanced autophagy” plays a role in cell death or represents a rescue mechanism with protective effects. Furthermore, whether autophagy can cause cell death directly or is a secondary effect of apoptosis remains to be determined. It should be noted that an increased number of autophagosomes is not always an indicator of enhanced autophagy. It may also indicate either an accumulation of uncleared autophagosomes due to impaired fusion with lysosomes, or a dysfunction in one of the various autophagy induction pathways.

**Figure 3:**
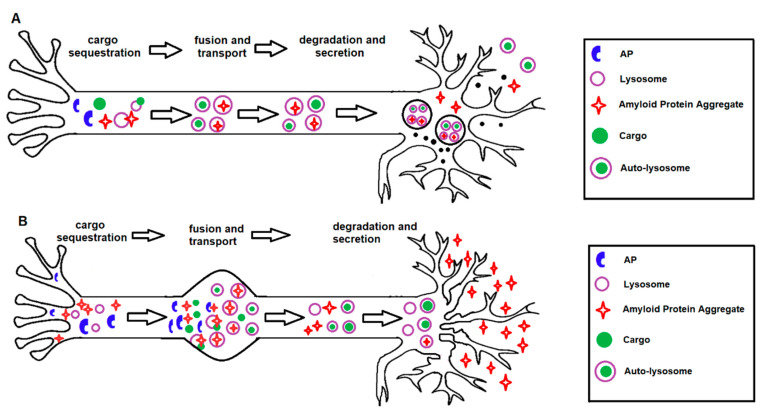
**Protective and detrimental effects of autophagy activation in AD.** (**A**) Under normal conditions, autophagy activation serves as a protective mechanism in healthy neurons with functional lysosomal clearance, by regulating the homeostasis of the axon terminal, membrane recycling, presynaptic function and removal of neurotoxins. (**B**) Under pathogenic conditions, as seen in human AD brains, autophagy activation can become detrimental in neurons with pre-existing protein aggregation and dysfunctional lysosomal clearance. In addition, autophagy activation could potentially lead to increased accumulation of immature autophagosomes, impaired autophagosome–lysosome fusion or may lead to the excessive degradation of essential organelles, self-digestion and neuronal death.

In conclusion, stimulation of autophagy in AD may have potential as a neuroprotective strategy, as long as excessive stimulation, which can be destructive, is avoided (Figure 3). It is essential to consider that modifying autophagy may lead to diverse consequences and interfere with mechanisms that are yet to be unravelled. By revealing the molecular mechanisms involved in autophagy and the role that this process plays in neuronal survival and death pathways, it should be possible to determine whether inhibition or stimulation of autophagy will be of therapeutic benefit in AD. From a therapeutic perspective, we believe that the evidence overall suggests that autophagy is a promising target. In addition to clearance of protein aggregates, autophagy upregulation has further protective effects by reducing the susceptibility to pro-death insults. One benefit of autophagy upregulation as a therapeutic approach is that constitutive activation of the pathway may not be required, as a targeted strategy to a particular component may be sufficient to improve autophagy efficacy.
